# Perceptual Load Affects Eyewitness Accuracy and Susceptibility to Leading Questions

**DOI:** 10.3389/fpsyg.2016.01322

**Published:** 2016-08-30

**Authors:** Gillian Murphy, Ciara M. Greene

**Affiliations:** ^1^School of Applied Psychology, University College CorkCork, Ireland; ^2^School of Psychology, University College DublinDublin, Ireland

**Keywords:** perceptual load, eyewitness memory, attention, perception, reconstructive memory

## Abstract

Load Theory (Lavie, 1995, 2005) states that the level of perceptual load in a task (i.e., the amount of information involved in processing task-relevant stimuli) determines the efficiency of selective attention. There is evidence that perceptual load affects distractor processing, with increased inattentional blindness under high load. Given that high load can result in individuals failing to report seeing obvious objects, it is conceivable that load may also impair memory for the scene. The current study is the first to assess the effect of perceptual load on eyewitness memory. Across three experiments (two video-based and one in a driving simulator), the effect of perceptual load on eyewitness memory was assessed. The results showed that eyewitnesses were less accurate under high load, in particular for peripheral details. For example, memory for the central character in the video was not affected by load but memory for a witness who passed by the window at the edge of the scene was significantly worse under high load. High load memories were also more open to suggestion, showing increased susceptibility to leading questions. High visual perceptual load also affected recall for auditory information, illustrating a possible cross-modal perceptual load effect on memory accuracy. These results have implications for eyewitness memory researchers and forensic professionals.

## Introduction

Selective attention is what allows us to focus on what is relevant and ignore irrelevant, potentially distracting information. It is what allows us to navigate complex environments and is critical for everyday functioning. This includes the ability to recall essential details after witnessing a crime—eyewitness memory. Load Theory (Lavie and Tsal, 1994; Lavie, 1995, 2005) makes specific predictions about the interplay of perception and awareness, stating that as perceptual capacity is limited, when perceptual load is high, irrelevant distractors are less likely to be processed. However, when a task incurs low perceptual load, all available stimuli are processed (including irrelevant distractors) and selective attention takes place at a later stage. Perceptual load is defined as “the amount of information involved in the processing of the task stimuli” (Macdonald and Lavie, 2011, p1780). This can be operationalized by either varying the number of task-related stimuli or by altering the task to be performed on the same stimuli. Load Theory has become a hugely influential model of attention and there is a body of evidence to suggest that high perceptual load reduces behavioral interference by irrelevant distractors (e.g., Lavie, 1995; Lavie and De Fockert, 2003; Forster and Lavie, 2008; but see Khetrapal, 2010; Benoni and Tsal, 2013; Cave and Chen, 2016 for reviews of recent studies challenging the load hypothesis). This theoretical approach builds directly on a large body of research in the field of selective attention, in particular efforts to resolve conflicting accounts of when in the attentional process distracting information is filtered out of awareness (see Murphy et al., 2016 for a review). Much of this evidence comes from research using variations of the Eriksen flanker task (Eriksen and Eriksen, 1974) in which interference from a peripheral distractor letter is reduced when the central task—a search array containing other letters—imposes high perceptual load. This corresponds with recent evidence that attention becomes more spatially focused under high perceptual load so that peripheral information is not processed to the same extent as central information (Caparos and Linnell, 2010).

Perceptual load does not just affect distraction by irrelevant stimuli; it also affects individuals’ subjective awareness of such stimuli. High load induces inattentional blindness, the phenomenon whereby people fail to notice easily visible stimuli (see Lavie et al., 2014 for a review). Cartwright-Finch and Lavie (2007) presented participants with a cross and asked them to either note which arm was blue (low load) or which arm was longer (high load), with the latter thought to consume considerably more attentional resources than the former, as identifying a color relies on discrimination of a single feature (Treisman and Gelade, 1980; Cartwright-Finch and Lavie, 2007). On a final, critical trial, an unexpected shape was also presented. Under high perceptual load only 10% of participants reported awareness for the shape, compared to 55% under low load. This effect of perceptual load on awareness is not limited to the visual domain; Macdonald and Lavie (2011) established the phenomenon of inattentional deafness, the failure to detect an auditory stimulus while engaged in a high visual load task. This suggests that perceptual load can also have cross-modal effects on attention and awareness.

Thus, there is much evidence to suggest that the processing of irrelevant information is reduced when a central task imposes high perceptual load. This has obvious implications for eyewitness memory, suggesting that memories for events that incur high perceptual load may be less accurate due to early attentional filtering. In a related study, Rivardo et al. (2011) manipulated attentional set and found that eyewitnesses who experienced inattentional blindness when watching a video of a theft were also less likely to recall other details from the video. There is some evidence for perceptual load effects on memory. In one study (Lavie et al., 2009), participants performed a low or high load letter search task while attempting to ignore salient but task-irrelevant distractors at fixation, such as a spider or a car. Recognition for the task-irrelevant object in a surprise test was significantly worse in the high load condition. Similar results have been found when human faces are used as irrelevant distractors (Jenkins et al., 2002, 2005). Though these studies suggest that perceptual load affects memory for task-irrelevant stimuli, there is as yet no evidence that this extends beyond artificial, computer-based search tasks. These studies also clearly defined the objects and faces as irrelevant and participants were instructed to ignore them. In a naturally occurring event, eyewitnesses may not be able to make a clear distinction between relevant and irrelevant information. There is evidence that real world situations which place high demands on attention can result in reduced memory accuracy and increased suggestibility to leading questions. This is true for when individuals are presented with attention-grabbing stimuli such as a weapon (the “weapon-focus effect”; Pickel, 1998; Saunders, 2009) and when they are instructed to perform a divided-attention task during encoding (Reinitz et al., 1994; Lane, 2006). However, as in the perceptual load examples above, these studies involve a specific manipulation (e.g., the presence of a weapon, completion of a secondary task) that affects the scope of attentional selection. Memory for details outside the attentional “spotlight” is therefore impaired. We argue that perceptual load is very different to a weapon focus effect as it is the content of the *entire* scene that maxes out all available attention capacity. When there is no obvious point around which to “tighten the spotlight,” how then will memory be affected by load? We hypothesize that load will detrimentally affect memory accuracy for all details but especially stimuli in the periphery, away from the main activity, due to the known spatial narrowing effects of high perceptual load (Caparos and Linnell, 2010).

Finally, we will investigate the effect of post-event information on low and high load memories. The effect of leading questions on eyewitness reports is well established (Loftus, 2003, 2005). For example, Loftus and Zanni (1975) showed participants a video of a car accident and asked either “Did you see *a* broken headlight?” or “Did you see *the* broken headlight?” The simple change from an indefinite to a definite article increased the rate of false positives from 7 to 18%. There is a wealth of evidence suggesting that eyewitness memory is malleable and open to suggestion. There is also evidence that individuals use other sources of information to aid in the reconstruction of memories, such as schemas and information contained in the question (Tuckey and Brewer, 2003; Loftus, 2005). As participants will be unable to take in all the details of a high load scene, we hypothesize that these memories will be less complete and individuals will therefore be more reliant on external cues to reconstruct their memory. This may lead to increased susceptibility to post-event misinformation and leading questions.

### The Present Study

In the research reported here, we explored three key questions. (1) Are eyewitness reports for high load events less accurate than for low load events? (2) Does high load induce spatial narrowing, so that peripheral details are especially affected by load? (3) Are high load memories more susceptible to the effects of leading questions than low load memories?

## Experiment 1A

### Method

#### Participants

During “Culture Night 2014,” an annual event in which museums and other places of interest are open to the public, 111 visitors (66 female) to University College Cork (UCC) School of Applied Psychology participated in this experiment (mean age = 34.37, *SD* = 13.89). No participants were excluded from analysis and none were added at a later date.

#### Materials and procedure

**FIGURE 1:**
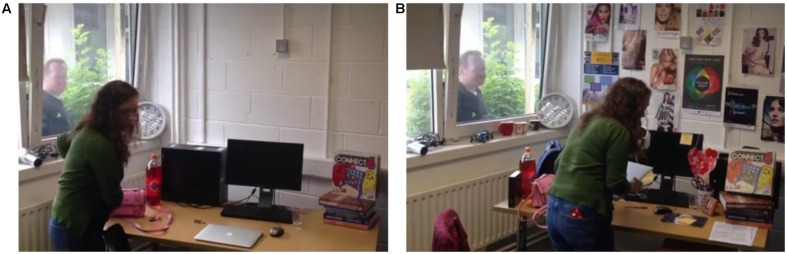
**Screenshots of the low perceptual load video **(A)** and the high perceptual load video **(B)**.** The thief is visible in the foreground and the witness is visible outside the window.

The study received ethical approval from UCC School of Applied Psychology Ethics Committee. All subjects gave written informed consent in accordance with the Declaration of Helsinki. Participants were randomly assigned to one of four conditions—high/low perceptual load video and regular/leading questionnaire. In small groups (*N*≈15), participants viewed a 1-min video featuring a robbery. They were instructed to pay close attention, as they would be questioned about the details. The video showed a woman entering an office and stealing a number of items. Midway through the video, a man walks past the window and looks into the office for approximately 5 s before walking off screen. There were two versions of this video, incurring either low or high perceptual load (see **Figure 1**). Perceptual load in the videos was manipulated via the number of objects in the scene (e.g., posters on the wall, objects on the desk, etc.). While the furniture remained constant between both videos, the low load video contained 13 additional items and the high load video contained 51 items, thus there are more features to be perceived and the load on attention is increased. The longer-term effects of load on eyewitness memory were assessed via a follow-up questionnaire, sent to participants via email 1 week after the experiment. However, in experiment 1A, the response rate was poor (38/111); these results are therefore not included here but can be found in Supplementary Material.

**FIGURE 2:**
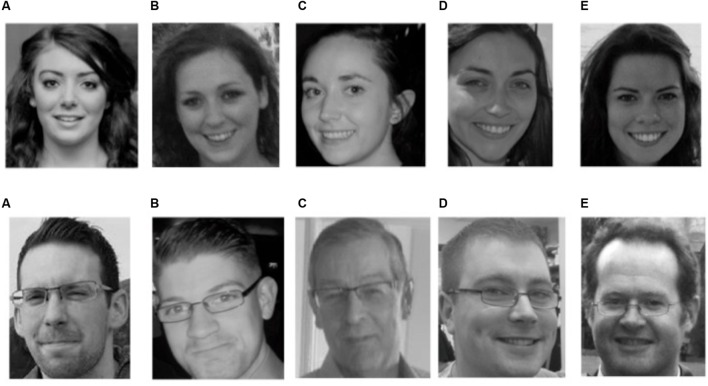
**Line-ups presented in questionnaires (Q4 and Q5) for experiment 1A and 1B.** The questions were worded “Please indicate if one of the above people was the thief/witness at the window in the video. Write A, B, C, D, E, or “none” if it was none of the above.” The correct answer for the thief (top row) is B and the correct answer for the witness (bottom row) is E. If participants failed to respond or wrote any other response, the question was marked as incorrect.

Immediately after viewing the video, participants completed a 22-item paper questionnaire, containing either regular or leading questions. Twelve were filler items; the 10 critical questions are listed in **Table 1**. Q1–Q6 tested memory for details of the video and were identical in both questionnaires; these included Q4, featuring a line-up containing the thief and four similar women, and Q5, featuring a line-up with the witness and four similar men (see **Figure 2**). The leading versions of Q7 and Q8 were designed to influence immediate responses (e.g., to imply there *was* a stapler) while Q9 and Q10 were designed to influence memory at follow-up (e.g., to imply that the thief was wearing a watch) and so responses to those questions were not used for analysis of memory accuracy.

**Table 1:** List of critical questions used in the regular, leading, and follow-up questionnaires in experiment 1A and 1B.

	Regular	Leading	Follow-up
Q1	What did the thief take?	–	
Q2	Did you notice any unusual objects on the desk?	–	
Q3	Did you notice any unusual objects on the windowsill?	–	
Q4	Thief line-up		
Q5	Witness line-up		
Q6	How confident are you that your memory for the event is accurate? (1–10)	–	F1. How confident are you that your memory for the event is accurate? (1–10)
Q7	Did you see a stapler on the desk?	Did you see the stapler on the desk?	F2. Did you see a stapler on the desk?
Q8	How long did the thief spend in the room? e.g., 5, 10, 15 s	How long did the thief spend in the room? e.g., 1, 2, 3 min	F3. How long did the thief spend in the room?
Q9	Was the thief wearing a watch?	Did the thief check the time on the watch they were wearing?	F4. Was the thief wearing a watch?
Q10	Did the thief look in the drawers?	Did the thief look in the drawers before or after taking the objects?	F5. Did the thief look in the drawers?


### Results

#### Perceptual Load

**FIGURE 3:**
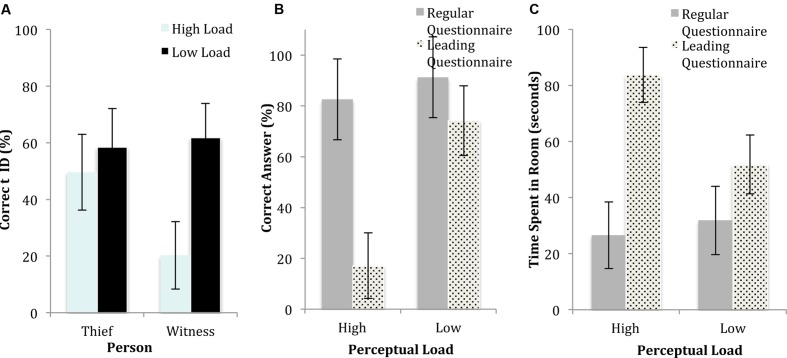
**Memory accuracy under low and high perceptual load in experiment 1A, with 95% confidence intervals shown.** **(A)** Accuracy of identification of the thief and the witness under low and high load. **(B)** Accuracy in response to the regular and leading stapler question (“*did you see the stapler*” vs. “*did you see a stapler*”) under low and high load. **(C)** Estimates of the time the thief spent in the room, in response to regular and leading questions under low and high load.

Participants were significantly less accurate under high load. Out of a total of three items stolen (laptop, money, and chocolate), participants correctly identified more items under low load [*M* = 2.45, 95% confidence intervals (CI_95_) = 2.24, 2.65] than high load [*M* = 1.48, CI_95_ = 1.29, 1.68; *F*(1,108) = 46.55, *p* < 0.001, *d* = 1.31]. CI_95_ are calculated as *M* ± 1.96 (SE) and are truncated at 0 and 100% where appropriate. Effect sizes are reported using Cohen’s *d* for main effects and eta squared for interactions. There were a number of unusual objects in the video, both on the desk in the center of the screen (a large bottle of soft drink, a game of Connect 4) and on the windowsill at the periphery (a toy car, a hairdryer). We defined a central detail as one that happens in the close vicinity of the main character (the thief), which is to say on the desk. We defined peripheral details as everything outside that area (including the windows, floor, etc.). Participants were required to list any items they remembered and answers were considered correct if one or more item was correctly reported. Accuracy for central objects (Q2) did not significantly differ between low load (*M* = 74.1%; CI = 61.3, 86.8) and high load [*M* = 65.5%, CI_95_ = 53.1, 77.9; *F*(1,108) = 0.92, *p* = 0.43]. However, load did have a significant effect on peripheral objects (Q3), with recall significantly better under low load (*M* = 49.8%, CI_95_ = 38.9, 60.7) relative to high load [*M* = 9.4%, CI_95_ = 0, 20; *F*(1,107) = 27.55, *p* < 0.001, *d* = 1.01]. Likewise, load had no effect on identification of the thief [low load: *M* = 58.4%, CI_95_ = 44.7, 72.2; high load: *M* = 49.6%, CI_95_ = 36.2, 63; *F*(1,108) = 0.82, *p* = 0.37] but did reduce identification of the witness at the window (see **Figure 3A**); low load (*M* = 61.7%, CI_95_ = 49.6, 73.8), high load [*M* = 20.3%, CI_95_ = 8.3, 32.2; *F*(1,107) = 23.44, *p* < 0.001, *d* = 0.93]. Load did not have a significant effect on participants’ tendency to falsely identify members of the line-up, with similar patterns of incorrect responses (% of participants choosing the wrong member of the line-up, participants indicating that the target was not present and participants indicating that they couldn’t choose/didn’t know) under low and high load. The precise distribution of responses is available in Supplementary Material. Participants rated themselves as marginally more confident in the accuracy of their memory (Q6) under low load (*M* = 5.46, CI_95_ = 4.92, 6) relative to high load (*M* = 4.56, CI_95_ = 4.04, 5.09), but this did not survive correction for multiple comparisons [*F*(1,108) = 5.59, *p* = 0.02].

#### Perceptual Load × Leading Questions

A significant interaction effect [*F*(1,108) = 10.66, *p* < 0.01, η^2^ = 0.9] showed that participants were less affected by the leading stapler question (Q7) under low load (see **Figure 3B**; regular: *M* = 91.3% correct, CI_95_ = 75.4, 100; leading: *M* = 74.2% correct, CI_95_ = 60.5, 87.9) compared to high load (regular: *M* = 82.6%, CI_95_ = 66.7, 98.5; leading: *M* = 17.1%, CI_95_ = 4.2, 30.1). As shown in **Figure 3C**, the same trend was evident with the misleading time-in-room question (Q8) with a reduced effect under low load (regular: *M* = 31.87 s, CI_95_ = 19.7, 44; leading: *M* = 51.8 s, CI_95_ = 41.32, 62.29) compared to high load [regular: *M* = 26.7 s, CI_95_ = 14.6, 38.9; leading: *M* = 83.7 s, CI_95_ = 73.9, 93.1; *F*(1,108) = 10.71, *p* < 0.01, η^2^ = 0.9].

## Experiment 1B

As response rates to the follow-up questionnaire were poor, experiment 1A was repeated with a larger sample of university students.

### Method

#### Participants

A total of 270 students (181 female) from two introductory psychology classes participated in this experiment during class time (mean age = 20.3, *SD* = 4.6). Participation was anonymous and participants were free to withdraw without penalty. All students present in class and willing to participate were included in the analysis and none were added at a later date. This study was approved by the UCC School of Applied Psychology Ethics Committee and all subjects gave written informed consent in accordance with the Declaration of Helsinki.

#### Materials and Procedure

The video and questionnaires were identical to experiment 1A.

### Results

Experiment 1B replicated the results of experiment 1A, finding significantly greater accuracy for Q1, items stolen, under low load (*M* = 2.47, CI_95_ = 2.32, 2.61) than high load [*M* = 1.26, CI_95_ = 1.14, 1.38; *F*(1,265) = 160.58, *p* < 0.001, *d* = 1.56]. Memory for items on the desk (Q2) did not differ significantly between low (*M* = 77.6%, CI_95_ = 69.7, 85.5) and high load [*M* = 76.4%, CI_95_ = 69.8, 83; *F*(1,265) = 0.06, *p* = 0.81], while objects on the windowsill (Q3) were recalled with significantly more accuracy under low (*M* = 85.7%, CI_95_ = 74.4, 97.1) relative to high load [*M* = 8.1%, CI_95_ = 0, 17.7; *F*(1,265) = 106.29, *p* < 0.001, *d* = 1.27]. In line with experiment 1A, there was no difference in the identification of the thief under low (*M* = 71.4%, CI_95_ = 62.6, 80.3) and high load [*M* = 62.3%, CI_95_ = 54.8, 69.7; *F*(1,265) = 2.43, *p* = 0.12], while the witness was correctly identified more often under low (*M* = 73.5%, CI_95_ = 65.1, 81.8) relative to high load [*M* = 28.9% CI_95_ = 21.9, 35.9; *F*(1,265) = 64.69, *p* < 0.001, *d* = 0.99]. Participants rated themselves as significantly more confident in the accuracy of their memory under low load (*M* = 5.65, CI_95_ = 5.31, 6.01) relative to high load [*M* = 4.64, CI_95_ = 4.35, 4.94; *F*(1,258) = 19.3, *p* < 0.001, *d* = 0.55].

#### Perceptual Load × Leading Questions

As in experiment 1A, participants were less affected by leading questions under low load but neither of these results survived correction for multiple (8) comparisons. Participants were less affected by the leading stapler question (Q7) under low load (regular: *M* = 83%, CI_95_ = 70.9, 95.1; leading: *M* = 75.9%, CI_95_ = 64.3, 87.4) compared to high load [regular: *M* = 58.1%, CI_95_ = 47.9, 68.4; leading: *M* = 28.2%, CI_95_ = 18.7, 37.8; *F*(1,266) = 4.19, *p* = 0.04, η^2^ = 0.02]. The same trend was evident with the misleading time-in-room question (Q8) with a reduced effect under low load (regular: *M* = 26.5 s, CI_95_ = 17.7, 35.3; leading: *M* = 76.1 s, CI_95_ = 67.8, 84.4) compared to high load [regular: *M* = 25.1 s, CI_95_ = 17.6, 32.6; leading: *M* = 96 s, CI_95_ = 89.1, 103; *F*(1,108) = 7.03, *p* = 0.008, η^2^ = 0.02].

#### Follow-Up

One hundred and ninety-one participants completed the follow-up questionnaire. Forty-five were in the high load-regular questionnaire condition, 53 were high load-leading questionnaire, 37 were low load-regular questionnaire and 56 were low load-leading questionnaire. Confidence in memory accuracy (F1) was not significantly affected by load at exposure: low load (*M* = 4.06, CI_95_ = 3.61, 4.51), high load [*M* = 3.65, CI_95_ = 3.22, 4.08; *F*(1,183) = 1.71, *p* = 0.19, *d* = 0.19].

Under low load, questionnaire type had no effect on correct answers to the stapler follow-up question; F2 (regular: *M* = 75.7% correct, CI_95_ = 62.7, 88.7; leading: *M* = 76.8%, CI_95_ = 66.2, 87.4) while under high load the effect was greater (regular: *M* = 31.1%, CI_95_ = 19.3, 42.9; leading: *M* = 7.5%, CI_95_ = 0, 18.4). The main effect of load was significant [*F*(1,187) = 93.48, *p* < 0.001, *d* = 1.41] but the interaction did not survive correction [*F*(1,187) = 4.39, *p* = 0.04, η^2^ = 0.02]. The same was true for the time-in-room question (F3), where there was a greater effect of leading question under high load (regular: *M* = 74.09 s, CI_95_ = 49.3, 98.9; leading: *M* = 165.38 s, CI_95_ = 142.3, 188.47) compared to low load (regular: *M* = 51.8 s, CI_95_ = 23.66, 79.94; leading: *M* = 89.46 s, CI_95_ = 67, 111.91). The main effect of load was significant [*F*(1,183) = 15.36, *p* < 0.001, *d* = 0.58] but the interaction again failed to survive correction [*F*(1,183) = 4.58, *p* = 0.03, η^2^ = 0.03].

For the question designed to implant false information regarding the watch (F4), there was no difference in the effect of the leading question under low load (regular: *M* = 78.4% correct, CI_95_ = 63.1, 93.7; leading *M* = 67.9% correct, CI_95_ = 55.4, 80.3) compared to high load (regular: *M* = 55.6% correct, CI_95_ = 41.7, 69.4; leading: *M* = 34% correct, CI_95_ = 21.2, 46.7). The main effect of load was significant [*F*(1,187) = 16.87, *p* < 0.01, *d* = 0.6] but the interaction was not [*F*(1,187) = 0.64, *p* = 0.42, η^2^ = 0.003]. Likewise for question F5 regarding the drawers, there was no difference in the effect of the leading question under low load (regular: *M* = 62.2% correct, CI_95_ = 46.4, 77.9; leading: *M* = 60.7% correct, CI_95_ = 47.9, 73.5) compared to high load (regular: *M* = 48.9% correct, CI_95_ = 34.6, 63.2; leading *M* = 28.3% correct, CI_95_ = 15.1, 41.5). The main effect of load was significant [*F*(1,187) = 10.3, *p* < 0.01, *d* = 0.47] but the interaction was not [*F*(1,187) = 1.81, *p* = 0.18, η^2^ = 0.002].

## Experiment 2

The purpose of experiment 2 was to extend the findings of experiment 1 to a more complex, realistic scenario using a driving simulator.

### Method

#### Participants

This study was also approved by the UCC School of Applied Psychology Ethics Committee. All subjects gave written informed consent in accordance with the Declaration of Helsinki. Experiment 2 was also conducted during Culture Night, and participants were 93 visitors to UCC (58 female), mean age = 35.9, *SD* = 13.2. All participants who completed the experiment were included in the analysis and none were added at a later stage. All participants had a driving license and an average of 15.8 years driving experience (*SD* = 13.2). Half the participants were driving and half were passengers in the front passenger seat. Participants were randomly assigned to one of four conditions—high/low perceptual load drive and regular/leading questionnaire.

#### Materials and Procedure

The experiment took place in UCC’s Driving Simulator Laboratory, featuring a five-door Volkswagen Polo with manual transmission. The simulator uses STISIM software^1^ and has a 180° forward view created by three floor-to-ceiling screens located 1.5 m from the car body. There is a screen behind the vehicle which projects a rear-view simulation and there are wing-mirror LCD display screens.

**FIGURE 4:**
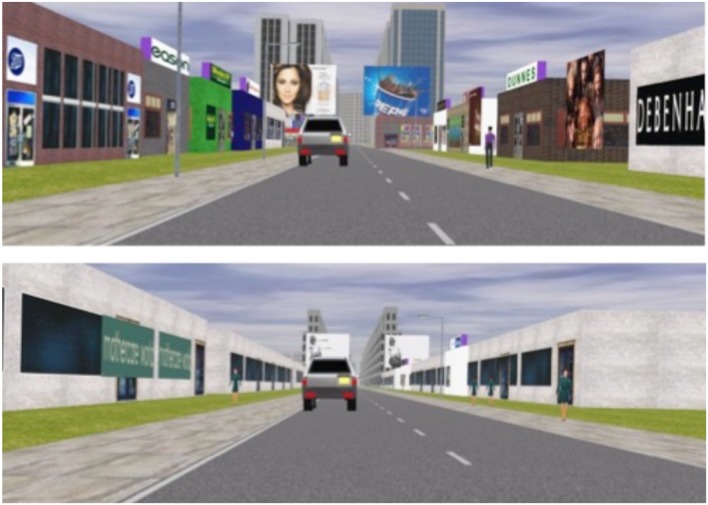
**Screenshots of the shopping area in the high load **(A)** and low load **(B)** driving tasks.** Pictured is the jeep that is about to be involved in the accident at the crossroads under the billboards.

Participants drove a 3 km route along a residential street that turned into a shopping area after approximately 2 km. Participants were instructed to pay close attention, as they would be asked questions immediately after the drive. As drivers entered the retail area a jeep pulled out behind them, drove behind them for approximately 10 s and overtook them. The jeep then crossed through a four-way intersection and collided with a red vehicle coming from the left. The noise of a car braking could be heard just before the crash, and the crash itself was accompanied by the sound of glass smashing. As the crash happened, a blue motorcycle approached the junction from the right, stopping before entering the intersection. Central and peripheral details were again defined as the area around the main characters in the scene (in this case the vehicle in front which is involved in an accident). All other details, including other vehicles outside of the intersection that were not involved in the crash, were considered peripheral. There were two versions of this drive; both featured the same buildings and vehicles and differed only in the surface detail of objects. While the high load drive featured colorful billboards, shop fronts, vehicles and multiple pedestrian models, the low load drive featured plain shop fronts, black and white billboards, silver vehicles and one pedestrian model (see **Figure 4**).

Immediately after the drive, participants completed a 21-item questionnaire; eight questions were used for analyses and the rest were filler questions (see **Table 2**). There were two versions of the questionnaire, regular and leading. Q1–Q4 tested memory for details of the video and were identical in both questionnaires. The leading versions of Q5 and Q6 were designed to influence participants’ estimates of speed and duration. The leading version of Q7 was designed to implant false information (i.e., that there *was* a stop sign) and the effect was measured via Q8, which was identical for all participants. All participants were sent a follow-up questionnaire by email 1 week after the experiment. However, as in experiment 1A, response rate was poor with just 34/93 responses received. Due to a lack of power, the results of the follow-up study are not included here but are available in the Supplementary Material.

**Table 2:** Regular and leading questions for experiment 2.

	Regular	Leading
Q1	Were there traffic lights above the intersection?	–
Q2	What was the vehicle to the right of the intersection?	–
Q3	Did you hear a car braking before or after the noise of the crash?	–
Q4	How confident are you that your memory for the event is accurate? (1–10)	–
Q5	How fast was the red car going when it made contact with the jeep?	How fast was the red car going when it smashed into the jeep?
Q6	How long was the jeep behind you before it overtook you? e.g., 5, 10, 15 s	How long was the jeep behind you before it overtook you? e.g., 1, 2, 3 min
Q7	How fast was the jeep going when it reached the intersection?	How fast was the jeep going when it ran the stop sign and reached the intersection?
Q8	Did you see a stop sign for the jeep?	


### Results

There was no effect of driver/passenger for any of the questions and so the data presented here are the combined results for all participants.

#### Perceptual Load

**FIGURE 5:**
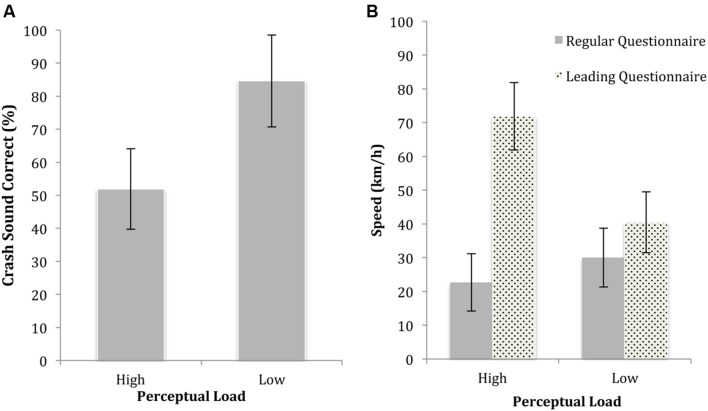
**Results from experiment 2 examining **(A)** cross-modal load effect of the low and high load drives, **(B)** the effect of a leading question about the speed of the red car, under low and high load**.

As shown in **Figure 5A**, load affected accuracy for the sound of braking (Q3), with more correct responses under low load (*M* = 84.6%, CI_95_ = 70.7, 98.4) than high load [*M* = 51.9%, CI_95_ = 39.7, 64.2; *F*(1, 89) = 12.32, *p* < 0.01, *d* = 0.74]. Load also significantly affected accuracy for peripheral details (Q2: the vehicle to the right of the intersection), with greater accuracy under low load (*M* = 45.2%, CI_95_ = 33.3, 57.2) than high load [*M* = 9.6%, CI_95_ = 0, 20.2; *F*(1,89) = 19.63, *p* < 0.001, *d* = 0.94]. However, there was no significant difference in accuracy for central details (Q1: traffic lights at the intersection) between low load (*M* = 82.3%, CI_95_ = 69.3, 95.3) and high load [*M* = 73.1%, CI_95_ = 61.6, 85; *F*(1,89) = 1.21, *p* = 0.29]. Confidence in memory accuracy (Q4) was not significantly affected by load at exposure: low load (*M* = 4.1, CI_95_ = 3.4, 4.8), high load [*M* = 4, CI_95_ = 3.3, 4.6; *F*(1,88) = 0.06, *p* = 0.81, *d* = 0.06].

#### Perceptual Load × Leading Questions

For the speed-of-the-red-car question (Q5), there was a greater effect of question type under high load (regular: *M* = 22.7 km/h, CI_95_ = 14.2, 31.3; leading: *M* = 71.9 km/h, CI_95_ = 63.2, 80.7) compared to low load [regular: *M* = 30 km/h, CI_95_ = 20, 40; leading: *M* = 40.5 km/h, CI_95_ = 31.5, 49.5; *F*(1,75) = 17.95, *p* < 0.001, η^2^ = 0.19], as shown in **Figure 5B**. For Q6, regarding the time the jeep was traveling behind them, there was a greater effect of the leading question under high load (regular: *M* = 7.4 s, CI_95_ = 4.4, 10.3; leading: *M* = 26.2 s, CI_95_ = 23.3, 29.1) compared to low load (regular: *M* = 7.8 s, CI_95_ = 4.7, 10.9; leading: *M* = 19.7 s, CI_95_ = 16.6, 22.8), however, this interaction effect did not survive correction for multiple comparisons [*F*(1,73) = 5.23, *p* = 0.03, η^2^ = 0.07]. Finally the stop-sign question (Q8) was not significantly affected by load, with similar responses under low load (regular: *M* = 15.8% false positives, CI_95_ = 0, 35.2; leading: *M* = 54.5% false positives, CI_95_ = 36.5, 72.6) and high load (regular: *M* = 30.8% false positives, CI_95_ = 14.2, 47.4; leading: *M* = 88.5% false positives, CI_95_ = 71.9, 100). The main effect of load was significant [*F*(1,89) = 7.52, *p* < 0.001, *d* = 0.58] but the interaction was not [*F*(1,89) = 1.13, *p* = 0.29, η^2^ = 0.01].

## Discussion

These experiments provide evidence that perceptual load affects eyewitness memory accuracy. High perceptual load events were recalled with less accuracy and these memories were more malleable, with recall strongly affected by leading questions. Experiments 1A and 1B provide evidence for these effects using a similar paradigm to most eyewitness memory research: a video of a simulated crime. The results of experiment 2 suggest that perceptual load may also affect memories for complex, real world events such as road traffic accidents. Interestingly, confidence ratings did not consistently differ for low and high load conditions across the three studies. This suggests that although participants in the high load condition were significantly less accurate in their recall, they were not necessarily aware of the deficit in their memory.

This study has clear implications for Load Theory, providing novel evidence that perceptual load affects everyday behavior. Scenes imposing high perceptual load consumed eyewitnesses’ attentional capacity and thus they could not take in every detail, resulting in less accurate memories and greater susceptibility to leading questions. Participants who had viewed the high perceptual load scenes were more likely to report the presence of items that were not in fact in the scene (e.g., a stapler or a stop sign) and their judgments of time and speed were more influenced by subtle suggestion. In line with our hypotheses, participants’ recall of peripheral stimuli (but not central stimuli) was significantly impaired under high perceptual load. This was true for both inanimate objects and for human faces, and is in line with previous research suggesting that perceptual load restricts the attentional spotlight (Caparos and Linnell, 2010).

Experiment 2 manipulated perceptual load by varying the complexity of objects along the route, rather than altering the task requirements for drivers. This load manipulation is simple and naturally occurring. Our findings suggest that eyewitness accounts for accidents on colorful, high perceptual load streets are likely less accurate and more open to suggestion. The cross-modal load effect observed in experiment 2 also has implications for driver safety and eyewitness reports. That such naturally occurring visual perceptual load impaired recall of a driving-relevant sound (the screech of sudden, sharp braking) suggests that it is not just visual attention that is affected by visual load. This is in line with “inattentional deafness” research (Macdonald and Lavie, 2011) but is the first evidence of such an effect in a realistic task.

One criticism of existing research on awareness under load is that experimenters typically use surprise post-trial questions about an unexpected or to-be-ignored stimulus. This raises the possibility that load effects arise as a result of rapid forgetting or “inattentional amnesia” (Wolfe, 1999) for the unexpected, weakly encoded stimulus. In the current experiments, participants were expressly told to pay attention to all details in the scene, as they would be questioned afterward and they were not asked to engage in any secondary task to divide their attention. This provides stronger support for Load Theory as the resulting perceptual load effects can be said to have arisen because participants had reached the limits of their attention capacity, not due to participants successfully ignoring stimuli that they have been told are irrelevant. This is important because there is a distinction between choosing to ignore irrelevant stimuli in a top-down fashion, and prioritizing central information because of bottom-up factors.

We have established the generality of our findings via different sets of rich materials, different load manipulations and by testing both university and general public samples, however, one limitation of this study is that the levels of load imposed by the two tasks were assumed to be low and high, and we intentionally used only materials depicting both extremes. As load is difficult to objectively quantify, we are essentially comparing *lower* load with *higher* load. But where our “low” and “high” load materials may rest on a continuum from absolute zero load to extremely high load is impossible to say. We do not know, for example, the exact point between a blank suburban street and a busy shopping area at which load begins to affect memory. Here, we implicitly assume a dichotomy based on previous research but in reality we do not know what effect moderate load may have on memory. Future research ought to examine the incremental transition from low to high load to further explore this issue.

One area where the issue of perceptual load may have a significant impact is in the case of child witnesses. Research has shown that young children demonstrate reduced memory accuracy and increased susceptibility to leading questions relative to adults (Goodman and Reed, 1986; Bruck and Ceci, 1999) and have a much smaller perceptual capacity that increases with age (Huang-Pollock et al., 2002; Couperus, 2011). The effect of perceptual load on attention may therefore have a disproportionate effect on children, and should be taken into consideration in future child memory studies.

As briefly mentioned in the introduction to this paper, there are valid criticisms of load theory and there are alternative hypotheses that seek to explain the results commonly found using load paradigms. Further research is needed to expand the scope of perceptual load research, particularly using ecologically valid paradigms, to assess the role of load in everyday attention. However, one competing theory, which is particularly relevant to the current study is that of attentional focus (see Cave and Chen, 2016 for a review). Chen and Cave (2016) have shown that when load is held constant, manipulating attentional focus can produce the same pattern of results seen in load experiments. Conversely, when attentional focus is held constant and load is manipulated, load effects are eliminated. Given the effect of the high load stimuli on memory for peripheral details in the current study, it is possible that attentional focus is an underlying factor. Future studies could attempt to isolate load and attentional focus to assess the effects on eyewitness memory. Similarly, future research could also examine eyewitnesses’ performance using eye-tracking technology to assess whether visual search strategies are significantly different under high load (i.e., that participants do not look at the male witness in the video) or whether the results are simply the effect of reduced attention capacity (i.e., participants “look but do not see”). This is an important distinction in terms of understanding the mechanisms driving the memory effects observed in the current study.

The current research proposes perceptual load as a novel factor in determining eyewitness accuracy and ability to withstand misinformation, despite not greatly reducing eyewitness’ confidence in their memory. Though it is possible to generate many applied forensic recommendations from these studies (e.g., that law enforcement officials ought to consider the level of visual and auditory load when evaluating eyewitness reports) most of these are difficult to implement given the current state of perceptual load literature. Load remains a subjective term that has not been objectively defined, and is therefore impossible to accurately measure (Murphy et al., 2016). As we cannot quantify load, it remains impossible to use the likely perceptual load of a scene to make inferences regarding the reliability or suggestibility of an eyewitness. However, we can conclude that perceptual load is an important factor in memory accuracy and eyewitness suggestibility. Though current efforts to use this finding in applied contexts are hampered by the lack of operational definitions, this area remains an exciting one, which may someday prove beneficial in the real world.

## Author Contributions

GM and CG developed the study concept and design. Data collection, analysis, and interpretation were performed by GM under the supervision of CG. GM wrote the manuscript and revised it in response to CG’s feedback. Both authors approved the final version of the manuscript for publication.

## Conflict of Interest Statement

The authors declare that the research was conducted in the absence of any commercial or financial relationships that could be construed as a potential conflict of interest.

## Footnotes

1 stisimdrive.com
