# Supplementary material for: Perceptual Load Affects Eyewitness Accuracy and Susceptibility to Leading Questions
Source: Front Psychol. 2016 Aug 30;7:1322. doi: 10.3389/fpsyg.2016.01322 (PMC5003837; doi:10.3389/fpsyg.2016.01322)
Supplement: Supplementary file 1 [file Data_Sheet_1.DOCX]

**Perceptual Load Affects Eyewitness Accuracy & Susceptibility to Leading Questions**

Gillian Murphy^1^ and Ciara M. Greene^2^

^1^University College Cork, ^2^University College Dublin

Online supplemental – line-up details and follow-up data for Experiment 1A and Experiment 2.

Corresponding Author

Gillian Murphy, University College Cork, Ireland.

[gillian.murphy@ucc.ie](mailto:gillian.murphy@ucc.ie)

**Experiment 1A**

Table S1: Responses to the thief/witness line-ups in Experiment 1A. Shown are the number of participants who correctly identified the target, who incorrectly selected one of the filler characters and those who stated that the target was not present. The final column (Unsure) represents ‘I don’t know’ responses as well as cases where participants selected more than one option.

|  | **Person** | **n** | **Target ID** | **Filler ID** | **Not Present** | **Unsure** |
| --- | --- | --- | --- | --- | --- | --- |
| ***Low Load*** | **Thief** | 54 | 31 | 19 | 4 | 0 |
|  | **Witness** | 54 | 32 | 14 | 8 | 0 |
|  | |  |  |  |  |  |
| ***High Load*** | **Thief** | 58 | 29 | 22 | 5 | 2 |
|  | **Witness** | 58 | 13 | 26 | 15 | 4 |

Table S2: List of critical questions used in the regular, leading and follow-up questionnaires in Exp. 1A.

|  | Regular | Leading | Follow-Up |
| --- | --- | --- | --- |
| Q1 | What did the thief take? | - |  |
| Q2 | Did you notice any unusual objects on the desk? | - |  |
| Q3 | Did you notice any unusual objects on the windowsill? | - |  |
| Q4 | Thief line-up |  |  |
| Q5 | Witness line-up |  |  |
| Q6 | How confident are you that your memory for the event is accurate? (1-10) | - | F1. How confident are you that your memory for the event is accurate? (1-10) |
| Q7 | Did you see a stapler on the desk? | Did you see the stapler on the desk? | F2. Did you see a stapler on the desk? |
| Q8 | How long did the thief spend in the room? e.g. 5, 10, 15 seconds. | How long did the thief spend in the room? e.g. 1, 2, 3 minutes. | F3. How long did the thief spend in the room? |
| Q9 | Was the thief wearing a watch? | Did the thief check the time on the watch they were wearing? | F4. Was the thief wearing a watch? |
| Q10 | Did the thief look in the drawers? | Did the thief look in the drawers before or after taking the objects? | F5. Did the thief look in the drawers? |

*Experiment 1A Follow-Up:* Follow-up questionnaires were sent via email one week after the experiment. Only participants who responded within 48 hours of were included in the results. This was to ensure that all responses would reflect memory as reported approximately one week after the event. For this experiment, 1 participant responded approximately 2 weeks after the deadline and so their data is not included here. There was a considerable attrition rate, with 38 out of 111 participants completing the follow-up questionnaire. 11 of these were in the high load-regular questionnaire condition, 11 were high load-leading questionnaire, 7 were low load-regular questionnaire and 9 were low load-leading questionnaire. Because of the very low power, the statistical analyses reported below ought to be interpreted with caution. The follow-up source confusion question was asked only in Experiment 1B and is therefore absent from the follow-up data for this experiment.

As can be seen in Table S1, there were 5 follow-up questions. Confidence in memory accuracy (F1) was affected by load at exposure: low load (M= 5.3, CI_95_ = [4.4, 6.3)), high load (M=3.8, CI_95_ = [3, 4.64]), (F (1, 34) = 6.12, p < .05, *d* = .39). Under low load, questionnaire type had no effect on correct answers to the stapler follow-up question; F2 (*regular:* M= 100% correct, CI_95_ = [71.9, 100]; *leading:* M=80%, CI_95_ = [56.5, 100]) while under high load the effect was greater (*regular:* M= 72.7%, CI_95_ = [50.3, 95.1]; *leading:* M= 9.1%, CI_95_ = [0, 31.5]). The main effect of load was significant (F (1, 35) = 16.94, p < .001, *d* = .57) but the interaction was not (F (1, 35) = 3.35, p = .08, η^2^ = .09). However, for the time-in-room question (F3), high load (*regular:* M= 90 seconds, CI_95_ = [48.8, 128.4]; *leading:* M= 76.4 seconds, CI_95_ = [36.1, 116.6]) was similar to low load (*regular:* M= 72.1 seconds, CI_95_ = [21.6, 122.6]; *leading:* M= 73.3 seconds, CI_95_ = [28.8, 117.9]) and the interaction effect was not significant (F (1, 34) = .1, p = .8, η^2^ = .003).

For the question designed to implant false information regarding the watch (F4), there was a greater effect of the leading question under low load (*regular:* M= 100% correct, CI_95_ = [73.6, 100]; *leading* M= 80% correct, CI_95_ = [57.9, 100]) compared to high load (*regular:* M= 81.8% correct, CI_95_ = [60.8, 100]; *leading:* M= 9.1% correct, CI_95_ = [0, 30.2]), F (1, 35) = 5.53, p < .05, η^2^ = .03). Finally, for Question F5 regarding the drawers, there was no difference in the effect of the leading question under low load (*regular:* M= 100% correct, CI_95_ = [69.1, 100]; *leading:* M= 60% correct, CI_95_ = [34.2, 85.8]) compared to high load (*regular:* M= 81.8% correct, CI_95_ = [57.2, 100]; *leading* M= 18.2% correct, CI_95_ = [0, 42.8]). The main effect of load was significant (F (1, 35) = 5.23, p < .05, *d* = .77) but the interaction was not (F (1, 35) = .81, p = .37, η^2^ = .02).

**Experiment 2**

Table S2: List of critical questions used in the regular, leading and follow-up questionnaires in Exp. 2.

|  | Regular | Leading | Follow-Up |
| --- | --- | --- | --- |
| 1 | Were there traffic lights above the intersection? | - | How confident are you that your memory is accurate? (1-10) |
| 2 | What was the vehicle to the right of the intersection? | - | How fast was the red car travelling? |
| 3 | Did you hear a car braking before or after the noise of the crash? | - | How long was the jeep behind you before it overtook you? |
| 4 | How confident are you that your memory for the event is accurate? (1-10) | - | Was there a stop sign for the jeep? |
| 5 | Did you see a yellow school bus? | Could the driver of the jeep’s view have been obstructed by the yellow school bus to the left? | Did you see a yellow school bus? |
| 6 | Did you see a woman with a dog at the crossroads? | Did the woman with the dog at the crossroads remain on the footpath? | Did you see a pedestrian with a dog at the crossroads? |
| 7 | How fast was the jeep going when it reached the intersection? | How fast was the jeep going when it ran the stop sign and reached the intersection? |  |
| 8 | How fast was the red car going when it made contact with the jeep? | How fast was the red car going when it smashed into the jeep? |  |
| 9 | How long was the jeep behind you before it overtook you? e.g. 5, 10, 15 seconds. | How long was the jeep behind  you before it overtook you?  e.g. 1, 2, 3 minutes. | |

*Experiment 2 Follow-Up:* All participants responded within 48 hours of the deadline and so no data has been discounted. Once again there was a high rate of attrition, with just 34 of 93 participants completing the follow-up questionnaire. 11 of these were in the high load-regular questionnaire condition, 7 were high load-leading questionnaire, 6 were low load-regular questionnaire and 10 were low load-leading questionnaire. As with Experiment 1A, the statistical analyses reported below ought to be interpreted with caution due to low statistical power.

The follow-up questions can be seen in Table S2. Confidence in memory accuracy (F1) was not affected by load at exposure: low load (M= 3.8, CI_95_ = [3.1, 4.6)), high load (M=3.2, CI_95_ = [2.5, 3.95]), (F (1, 30) = .11, p = .74, *d* = .13). Under low load, questionnaire type had no effect on correct answers to the speed-of-the-red-car follow-up question; F2 (*regular:* M= 33.3 km/h, CI_95_ = [19.9, 46.8]; *leading:* M= 35 km/h, CI_95_ = [24, 6]) while under high load the effect was much greater (*regular:* M= 36.8 km/h, CI_95_ = [26.9, 46.7]; *leading:* M= 75.1 km/h, CI_95_ = [63.3, 88.2]), F (1, 29) = 10.45, p < .01, η^2^ = .26. However, for the time-jeep-was-behind question (F3), high load estimates (*regular:* M= 28.6 seconds, CI_95_ = [11.8, 45.5]; *leading:* M= 62.9 seconds, CI_95_ = [41.7, 84]) and low load estimates (*regular:* M= 8.3 seconds, CI_95_ = [0, 31.2]; *leading:* M= 21.4 seconds, CI_95_ = [2.8, 40.1]) did not have a significant interaction effect (F (1, 29) = 1.17, p = .29, η^2^ = .04).

For the question designed to implant false information regarding the stop sign (F4), there was a greater likelihood of false positives in response to the leading question under high load (*regular:* M= 18.2% false positives, CI_95_ = [0, 43.1]; *leading* M= 85.7%, CI_95_ = [54.4, 100]) compared to low load (*regular:* M= 16.7%, CI_95_ = [0, 50.5]; *leading:* M= 20%, CI_95_ = [0, 46.2]), F (1, 30) = 5.01, p < .05, η^2^ = .14). For Question F5 regarding the yellow school bus, there was a significant interaction between load and questionnaire type: low load (*regular:* M= 16.7% false positives, CI_95_ = [0, 48]; *leading:* M= 10%, CI_95_ = [0, 34.2]) compared to high load (*regular:* M= 18.2%, CI_95_ = [0, 41.3]; *leading* M= 85.7% correct, CI_95_ = [56.7, 100]), F (1, 30) =7.81, p < .01, η^2^ = .21. Finally, for Question F6 regarding the dog, there was no difference in the effect of the leading question under low load (*regular:* M= 0% false positives, CI_95_ = [0, 33.6]; *leading:* M= 10%, CI_95_ = [0, 36]) compared to high load (*regular:* M= 36.4%, CI_95_ = [11.5, 61.2]; *leading* M= 71.4%, CI_95_ = [40.3, 100]). The main effect of load was significant (F (1, 30) = 11.77, p < .01, *d* = .77) but the interaction was not (F (1, 30) = .77, p = .39, η^2^ = .03).
